# Case report: Intraneural perineurioma in dogs: a case series and brief literature review

**DOI:** 10.3389/fvets.2023.1233230

**Published:** 2024-01-11

**Authors:** Ji-Hang Yin, Brittani Sexton, Tom Jukier, Amy B. Yanke, Merrilee Holland, Andrew D. Miller, Lauren Stranahan, Aline Rodrigues Hoffmann, Maninder Sandey

**Affiliations:** ^1^Department of Pathobiology, College of Veterinary Medicine, Auburn University, Auburn, AL, United States; ^2^Department of Clinical Sciences, College of Veterinary Medicine, Auburn University, Auburn, AL, United States; ^3^Department of Population Medicine and Diagnostic Sciences, Section of Anatomic Pathology, College of Veterinary Medicine, Cornell University, Ithaca, NY, United States; ^4^Department of Veterinary Pathobiology, Texas A&M School of Veterinary Medicine & Biomedical Sciences, Texas A&M University, College Station, TX, United States

**Keywords:** canine, spinal nerve roots, intraneural perineurioma, immunohistochemistry, brief literature review

## Abstract

Intraneural perineurioma is an exceptionally rare neoplasm in animals. This case study comprises a series of three cases and a brief literature review focusing on canine intraneural perineurioma. The pathological and immunohistochemical findings are documented, revealing that canine intraneural perineurioma frequently affects adult dogs aged between 3 and 10 years old, with a male predominance. Clinical signs associated with intraneural perineurioma in dogs include spinal pain, lameness, and paresis, resulting from the involvement of spinal nerve roots of the pelvic limbs, brachial plexus, or distal part of the median nerve. Most neoplasms had characteristic pseudo-onion bulb patterns on histopathology. Neoplastic perineurial cells, in most cases, expressed laminin and claudin-1, and NF200 consistently highlighted the central axon. While the immunohistochemical (IHC) profile of intraneural perineurioma in veterinary medicine remains incompletely characterized, the available IHC data from all reported cases suggest that a combination of laminin and claudin-1 immunomarkers, along with distinctive histological features, can assist in establishing a definitive diagnosis of intraneural perineurioma.

## Introduction

The World Health Organization (WHO) classification of tumors of the peripheral nerves includes Schwannoma, neurofibroma, perineurioma, and malignant peripheral nerve sheath tumors (1). Among these, perineurioma is rarely reported in the human literature and has limited cases documented in veterinary medicine (2–8).

Perineurioma is a benign and slow-growing neoplasm composed exclusively of perineurial cells (9, 10). In human medicine, two variants of perineuriomas are recognized: extraneural soft tissue tumor and intraneural variant (1). The extraneural soft tissue tumors are typically well-demarcated, unencapsulated masses that most commonly develop in subcutaneous tissues of the extremities or trunk and are rarely associated with an identifiable nerve (1, 11). Conversely, an intraneural perineurioma usually develops as a solitary nodular mass that commonly involves peripheral nerves or nerve roots of the cervical and lumbar spinal cord (1, 11).

Despite the distinct affected sites, both variants share immunohistochemical and ultrastructural features of neoplastic perineurial cells (1, 10). Histologically, intraneural perineurioma contains characteristic “pseudo-onion bulbs” structures composed of concentric layers of perineurial cells surrounding either myelinated or non-myelinated axons or endoneurial capillaries (1). Neoplastic perineurial cells are spindle-shaped cells with a fusiform to elongate nucleus and a scant amount of eosinophilic cytoplasm, histomorphologically resembling fibroblasts. Neoplastic perineurial cells can be distinguished from fibroblasts by electron microscopy. Ultrastructurally, normal and neoplastic perineurial cells have elongated, thin, and overlapping cytoplasmic processes, discontinuous basal lamina, and pinocytocytic vesicles (9). Some overlapping processes are connected by poorly formed desmosome-like junction complexes (5, 6, 9, 12).

In veterinary medicine, diagnosing various subtypes of benign peripheral nerve sheath tumors, such as Schwannoma, neurofibroma, and perineurioma, can be challenging due to shared morphological features. Schwannomas are benign and well-circumscribed tumors, consist of neoplastic Schwann cells arranged in diverse patterns like interwoven bundles, streams, herringbone, whorls, or Verocay bodies. In contrast, neurofibromas comprise a mixed population of neoplastic Schwann cells, perineurial cells, and fibroblasts, separated by abundant collagen. Intraneural perineuriomas typically exhibit perineurial cells organized in pseudo-onion bulb structures, as previously described (7).

Various immunomarkers have been investigated to differentiate among different subtypes of peripheral nerve tumors. For example, laminin is commonly utilized in diagnosing peripheral nerve sheath tumors, although it is expressed in both neoplastic Schwann and perineurial cells. Sox-10, which exhibits high expression in Schwann cells, demonstrates strong and diffuse immunoreactivity in Schwannomas (14). NF200 and Periaxin are two immunomarkers that can be used to highlight centrally entrapped axons in pseudo-onion bulbs in perineuriomas (7, 14). In human medicine, epithelial membrane antigen (EMA) is a widely used immunohistochemical marker for identifying perineuriomas (9, 10, 12, 13); however, its applicability in veterinary species is limited. Additionally, claudin-1 and glucose transporter 1 (GLUT-1) are routinely used as supportive markers for perineurioma (10). In this study, we present three cases involving canine intraneural perineurioma. We documented signalment, clinical history, and detailed pathological and immunohistochemical findings for these cases. Additionally, we conducted a comprehensive review of previously reported cases and offered a concise literature review on canine intraneural perineurioma.

## Case 1

A 3-year-old male castrated beagle dog was referred to the Neurology and Neurosurgery Service at the Auburn University Veterinary Teaching Hospital due to chronic left pelvic limb lameness that had commenced 5 months before presentation.

Multiple magnetic resonance imaging (MRI) revealed a large, well-defined fusiform-shaped mass within the left caudal lumbar spinal canal. This mass extended from the caudal endplate of the L5 vertebra to the mid-vertebral body of L7, causing severe lateral displacement and compression of the L5-L7 spinal cord segments. These findings were consistent with neoplastic growth, including possible differentials such as meningioma, intradural peripheral nerve sheath tumor, and round cell neoplasia. Given the progression of the dog's clinical symptoms, the owner opted for humane euthanasia, and the dog underwent postmortem evaluation.

**Figure 1:**
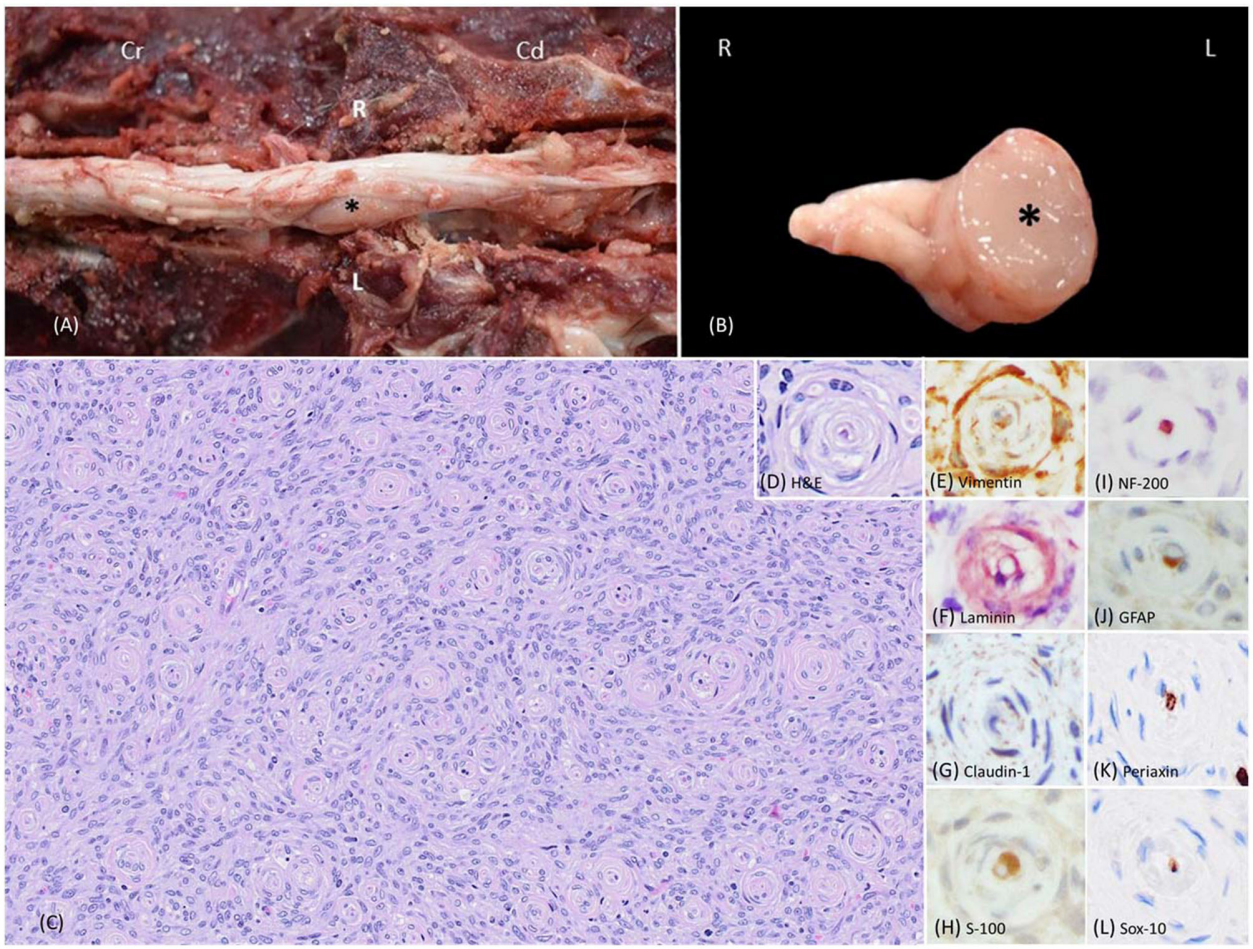
Images of intraneural-extramedullary perineurioma involving the left L5 spinal nerve roots in a beagle dog (case 1). **(A)** Gross photos show the spinal cord and an intraneural-extramedullary mass. The picture, taken post-dura opening, reveals an enlarged left spinal nerve root entrapped by a well-defined, unencapsulated, firm, tan mass measuring 2 cm x 0.5 cm x 0.5 cm (black star). Cr: Cranial; Cd: Caudal. L: left-sided; R: right-sided. **(B)** The cross-section of the intraneural-extramedullary mass (black star). L: left-sided; R: right-sided. **(C)** Histologically, the mass contained numerous pseudo-onion bulbs, characterized by neoplastic perineurial cells arranged in concentric lamellations wrapping a central myelinated or non-myelinated axon. Haematoxylin and eosin stain (H&E), 20X. **(D)** Higher magnification of the pseudo-onion bulbs of intraneural-extramedullary perineurioma. H&E, 40X. **(E)** Neoplastic perineurial cells had a strong cytoplasmic immunolabeling for vimentin antigen. Immunohistochemistry (IHC), DAB, 40X. **(F)** Neoplastic perineurial cells had a strong cytoplasmic immunolabeling for laminin antigen. IHC, Vector NovaRED, 40X. **(G)** Neoplastic perineurial cells had a strong cytoplasmic immunolabeling for claudin-1 antigen. IHC, DAB, 40X. **(H)** The Schwann cells were immunolabeled with S-100, while the neoplastic perineurial cells lacked immunoreactivity. IHC, DAB, 40X. **(I)** The central axon showed strong immunolabeling for the NF200 antigen. IHC, Vector NovaRED, 40X. **(J)** The Schwann cells were immunolabeled with GFAP, but the neoplastic perineurial cells lacked immunoreactivity. IHC, DAB, 40X. **(K)** The central axon showed immunolabeling for Periaxin antigen. Neoplastic perineurial cells lacked immunolabeling for Periaxin antigen. IHC, DAB, 40X. **(L)** The Schwann cells were immunolabeled with Sox-10 antigen. Neoplastic perineurial cells lacked immunolabeling for the Sox-10 antigen. IHC, DAB, 40X.

At necropsy, the left L5 spinal nerve root was enlarged, and a well-demarcated, unencapsulated, firm, tan, 2 x 0.5 x 0.5-cm mass that markedly compressed the adjacent L5-L7 spinal cord was identified (Figures 1A, B). Histologically, the mass was unencapsulated, mildly infiltrative, densely cellular, and composed of fusiform cells arranged in concentric lamellations that ensheathed a central axon or capillaries forming pseudo-onion bulbs structures (Figures 1C, D). Neoplastic cells had indistinct cell borders and a scant amount of eosinophilic cytoplasm. Nuclei were oval to elongate with finely stippled chromatin and 1-3 small nucleoli. The neoplastic cells had mild anisocytosis and anisokaryosis with four mitotic figures in ten standardized 400 x fields (2.37-mm^2^). All the formalin-fixed paraffin-embedded tissues of canine perineuriomas were subjected to immunohistochemistry staining (Supplementary Table 2). Immunohistochemically, approximately 90% of the neoplastic perineurial cells had strong and diffuse cytoplasmic immunolabeling for vimentin (Figure 1E) and laminin (Figure 1F), while approximately 50% had claudin-1 immunolabeling (Figure 1G). S-100 (Figure 1H) and glial fibrillary acidic protein (GFAP) immunolabeling was only observed in the Schwann cells within the pseudo-onion bulbs, in which multiple ensheathed axons were immunolabeled with neurofilament 200 (NF200) (Figures 1I,J). Neoplastic cells lacked immunoreactivity for Periaxin (Figure 1K) and Sox-10 (Figure 1L) antigens. Similar to NF200, Periaxin immunolabeling was detected in axons. In addition, Schwann cells within the pseudo-onion bulbs were highlighted with Sox-10 antibody.

## Case 2

A 10.7-year-old neutered male Labrador Retriever mix dog presented to a referral veterinary neurology clinic due to a two-week history of pelvic limb weakness. MRI scans revealed a strongly contrast-enhancing mass within the spinal cord extending along the right side of the spinal cord from C6-T1 and into the associated nerve roots. Clinical differentials included a malignant peripheral nerve sheath tumor or lymphoma. Due to the poor long-term prognosis, the patient was euthanized, and a postmortem examination was conducted.

**Figure 2:**
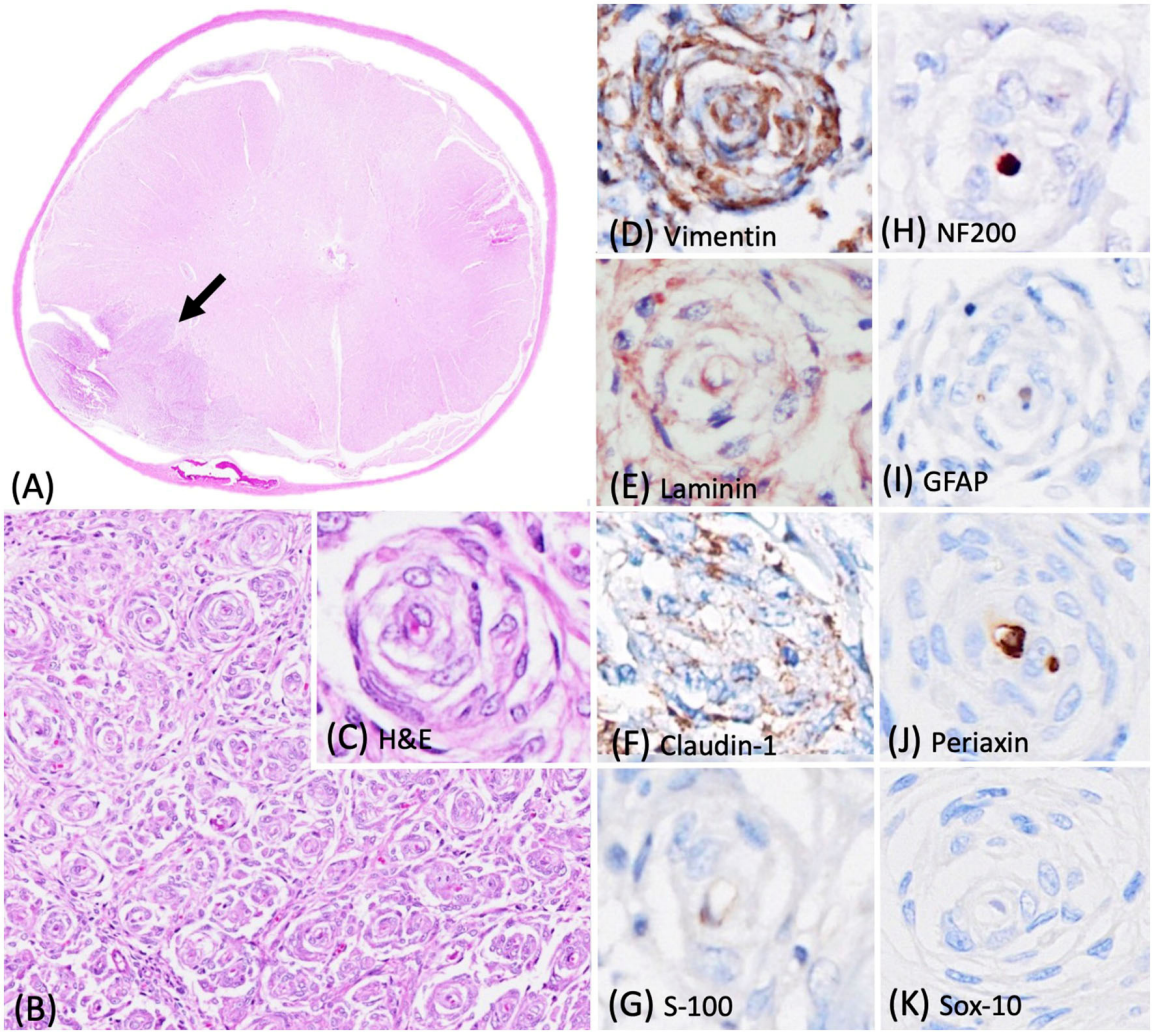
Photomicrographs of the intraneural-extramedullary perineurioma in a Labrador Retriever mix dog (case 2). **(A)** The lateral to anterior funiculus level of the right-sided spinal cord at C6-T1 contained a spinal mass with numerous pseudo-onion bulbs, characterized by neoplastic perineurial cells arranged in concentric lamellations wrapping a central myelinated or non-myelinated axon. Haematoxylin and eosin stain (H&E), 2X. **(B, C)** Higher magnification of the pseudo-onion bulbs of intraneural-extramedullary perineurioma. H&E, 20X and 40X. **(D)** Neoplastic perineurial cells had a strong cytoplasmic immunolabeling for vimentin antigen. Immunohistochemistry (IHC), DAB, 40X. **(E)** Neoplastic perineurial cells had a strong cytoplasmic immunolabeling for laminin antigen. IHC, Vector NovaRED, 40X. **(F)** Neoplastic perineurial cells had a fine punctate immunolabeling pattern for claudin-1 antigen. IHC, DAB, 40X. **(G)** The Schwann cells were immunolabeled with S-100, but the neoplastic perineurial cells lacked immunreactivity. IHC, DAB, 40X. **(H)** The central axon showed strong immunolabeling for the NF200 antigen. IHC, Vector NovaRED, 40X. **(I)** The Schwann cells were immunolabeled with GFAP, but the neoplastic perineurial cells lacked immunoreactivity. IHC, DAB, 40X. **(J)** The central axon showed immunolabeling for Periaxin antigen. Neoplastic perineurial cells lacked immunolabeling for Periaxin antigen. IHC, DAB, 40X. **(K)** Neoplastic perineurial cells lacked immunolabeling for the Sox-10 antigen. IHC, DAB, 40X.

During necropsy, a mass of approximately 0.7 cm in diameter effacing the right-side of the spinal cord was identified. The formalin-fixed spinal mass was submitted to Cornell University Animal Health Diagnostic Center for histopathologic diagnosis. Histologically, at the lateral to anterior funiculus level of the right-sided spinal cord at C6-T1, an unencapsulated, well-demarcated, and densely cellular neoplasm was observed, which multifocally compressed and invaded the adjacent spinal cord (Figure 2A). The neoplasm contained numerous pseudo-onion bulbs structures similar to those observed in the first case (Figures 2B, C). Immunohistochemical profiling of the neoplasm revealed that approximately 90% of the neoplastic perineurial cells were immunolabeled for vimentin (Figure 2D) and laminin (Figure 2E). Similar to that described in case 1, approximately 20% of the neoplastic perineurial cells showed a fine punctate immunolabeling pattern for claudin-1 antigen (Figure 2F). Multiple pseudo-onion bulbs contained centrally located NF200 immunolabeled axons ensheathed by GFAP and S-100 positive Schwann cells (Figures 2G–I). No immunolabeling was observed in neoplastic perineurial cells for Periaxin (Figure 2J) and Sox-10 (Figure 2K) antigens with multiple pseudo-onion bulbs contain centrally located Periaxin immunolabeled.

## Case 3

A 10-year-old spayed female Labrador Retriever dog presented to the Texas A&M Oncology Service with a four-month history of limping and lameness in the left thoracic limb. MRI revealed enlarged left spinal nerve roots at the C7-C8 as well as the right C8 nerve root. Clinical differentials included peripheral nerve sheath tumors or neuritis. The left C7 spinal nerve root was surgically removed for histopathologic evaluation.

**Figure 3:**
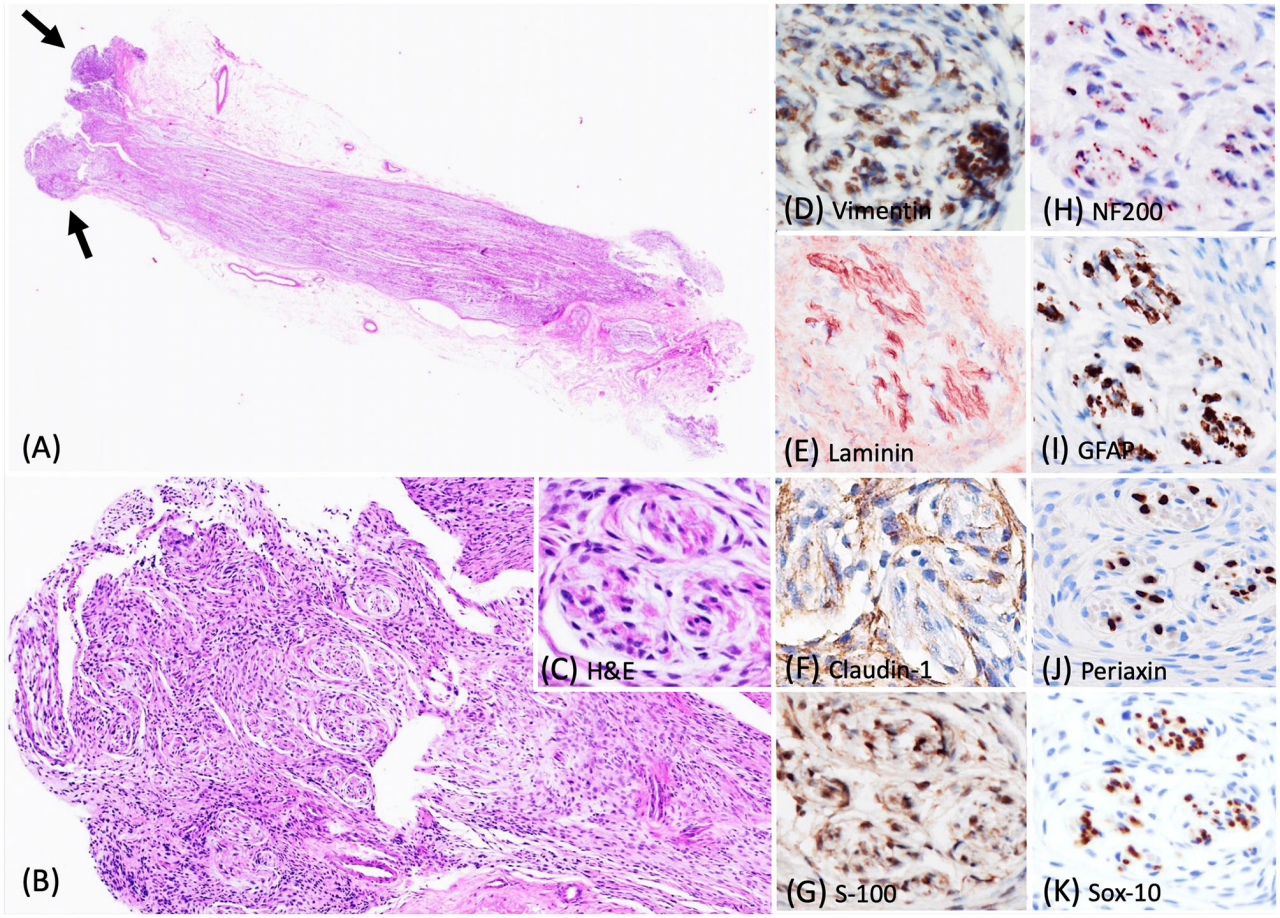
Photomicrographs of the intraneural-extramedullary perineurioma in a Labrador Retriever dog (case 3). **(A)** Histologically, the biopsied C7 spinal nerve root was mildly expanded by a unencapsulated mass, characterized by neoplastic perineurial cells arranged in concentric lamellations wrapping a central myelinated or non-myelinated axon (arrow). Haematoxylin and eosin stain (H&E), 2X. **(B, C)** Higher magnification of the pseudo-onion bulbs of intraneural-extramedullary perineurioma. H&E, 10X and 40X. **(D)** Neoplastic perineurial cells had a strong cytoplasmic immunolabeling for vimentin antigen. IHC, DAB, 40X. **(E)** Neoplastic perineurial cells had a strong cytoplasmic immunolabeling for laminin antigen. IHC, Vector NovaRED, 40X. **(F)** Neoplastic perineurial cells were immunolabeled with claudin-1 antigen. IHC, DAB, 40X. **(G)** Neoplastic perineurial cells had a strong cytoplasmic immunolabeling for S-100 antigen. IHC, DAB, 40X. **(H)** The central axon had a strong immunolabeling for the NF200 antigen. IHC, Vector NovaRED, 40X. **(I)** The Schwann cells were immunolabeled with GFAP, but the neoplastic perineurial cells lacked immunoreactivity. IHC, DAB, 40X. **(J)** The central axon showed immunolabeling for Periaxin antigen. Neoplastic perineurial cells lacked immunolabeling for Periaxin antigen. IHC, DAB, 40X. **(K)** The Schwann cells were immunolabeled with Sox-10 antigen. Neoplastic perineurial cells lacked immunolabeling for the Sox-10 antigen. IHC, DAB, 40X.

Histologically, the biopsied C7 spinal nerve root was mildly expanded by an unencapsulated, approximately 0.3 cm in diameter, densely cellular mass confined to the perineurium (Figure 3A). This mass contained a neoplastic population of spindle cells arranged in short, tight, haphazard streams, bundles, and whorls on the pre-existing stroma (Figures 3B, C). This pattern of the neoplastic proliferations closely resembled those of the previously reported canine perineuriomas (6). Approximately 90% of the neoplastic perineurial cells had immunolabeling for vimentin (Figure 3D) and laminin antigens (Figure 3E). Approximately 30–60% of the neoplastic perineurial cells showed fine punctate immunolabeling for claudin-1 antigen as that observed in case 1 (Figure 3F). Approximately 90% of the neoplastic perineurial cells in this case expressed S100 (Figure 3G). Multiple neoplastic whorls contained NF200 immunolabeled axons, often rimmed by GFAP labeled Schwann cells (Figures 3H, I). Similar to case 1 and 2, the neoplastic cells had negative immunolabeling for Periaxin (Figure 3J) and Sox-10 (Figure 3K) antigens.

## Discussion

In this case study, we described a case series of canine intraneural perineurioma and focused on the pathological and immunohistochemistry findings. Additionally, in our search across databases such as PubMed, CAB Direct, Web of Science, and Google Scholar, we found three previously reported cases of canine intraneural perineuriomas (Table 1). In summary, intraneural perineurioma commonly occurred in adult dogs, typically aged between 3 and 10 years in both small and large breeds. Most cases of canine perineurioma were observed in males (6/6). Clinical manifestations associated with intraneural perineurioma included symptoms such as spinal pain, lameness, and paresis, resulting from the involvement of spinal nerve roots or peripheral nerves. In all cases, large masses in the spinal canal or spinal roots were demonstrated in MRI and common gross findings included the presence of a cylindrical mass associated with nerve bundles. Histologically, most perineuriomas have neoplastic perineurial cells arranged in characteristic pseudo-onion bulb structures or whorls.

**Table 1:** Signalment, clinical history, anatomic location, and gross findings of reported canine intraneural perineurioma.

**Breed**	**Age (years)**	**Sex**	**Clinical signs**	**Location**	**Gross findings**	**References**
**Published cases**						
German shepherd	6	MN	Progressive, nonpainful, left pelvic limb paresis	Left L5-L6 spinal nerve roots	Focally and cylindrically thickened nerve roots (twice the normal size)	(6)
Beagle	9	M	Progressive, cervicothoracic pain, tetraparesis	Left brachial plexus	Cylindrical enlargement (0.8-cm in diameter)	(8)
Leonberger	4	M	2-year history left thoracic limb lameness	Distal part of median nerve	Markedly thickened distal part of median nerve (1 cm in diameter)	(5)
N/A	N/A	N/A	N/A	Cervical nerve root	N/A	(14)
Rottweiler	9	MN	Progressive, mild ataxia in all limbs	Left C1-2 spinal nerve root	Contrast enhancing intradural extramedullary mass at nerve root	(14)
Labrador retriever N/A	5	FS	Intermitent, 6-month, thoracic limb lameness/tetraparesis, pain	Left C2 nerve root	Contrast enhancing intradural intramedullary mass at nerve root (2 cm in diameter)	(14)
**Unpublished data**						
Labrador retriever	10	MN	Non-progressive, non-painful, hind limb weakness	C6-T1 spinal nerve root	Unencapsulated mass (0.7-cm in diameter)	Cornell University, unpublished data
Mixed breed dog	10	FS	4-month history limping and lameness on left forelimb	C7 left spinal nerve	Unencapsulated mass (0.3-cm in diameter)	Texas A&M University, unpublished data
Beagle	3	MN	5-month progressive pelvic limb weakness, lumbar pain	Left spinal root containing the spinal nerves of L4-L6	Unencapsulated mass (0.5-cm in diameter)	Auburn University, unpublished data

L, Lumbar; M, Male; MN, Male neutered.

The expression of various immunomarkers in nerve sheath tumors exhibits marked overlap, and the diagnostic utility of immunohistochemistry has not been definitively established. Several immunomarkers have been frequently employed in an effort to identify perineurioma and distinguish them from other nerve sheath tumors in both human and veterinary medicine. Various immunomarkers, including claudin-1, collagen IV, epithelial membrane antigen (EMA), GFAP, GLUT-1, NF200, neurofilament, periaxin-1, Sox-10, and S-100 antibodies, were used in these cases. In our presented cases (case 1–3), the neoplastic perineurial cells were all immunolabeled for vimentin, laminin, and claudin-1. NF200 and Periaxin consistently highlighted the central axon in three cases. Only one case (case 2) had S-100 labeling in the neoplastic perineurial cells. Similar to the published cases, vimentin (2/2 cases), laminin (5/5 cases), and claudin-1(1/1 case) frequently labeled neoplastic perineurial cells. Previously, Sisó et al. (14) has shown positive expression of S-100, GFAP, Periaxin, and Sox-10 in canine perineuriomas.

The intermediate filament protein GFAP, primarily expressed in glial cells, exhibits variable expression in benign nerve sheath tumors (14). No GFAP expression was detected in the presented cases 1–3. Laminin proteins are expressed in the basement membrane and has been utilized to identify perineurial cells and Schwann cells (15–17). Neoplastic perineruial cells in all presented cases had positive immunoreactivity for laminin. Claudin-1, primarily found in the tight junctions of endothelial and epithelial cells, is also expressed in both normal and neoplastic human perineurial cells (5, 18). Notably, ~92% of human perineuriomas have positive immunolabeling for claudin-1 (5). In dogs, claudin-1 is strongly expressed in both normal and neoplastic perineurial cells (5). Moreover, claudin-1 is absent in Schwann cells, making it a valuable tool for excluding other peripheral nerve neoplasms (5). In all cases, neoplastic perineurial cells had positive immunolabeling for claudin-1 in our study. Similarly, claudin-1 expression was also detected in a previously reported case of intraneural perineurioma in a 4-year-old male leonberger (5). However, claudin-1-poistive perineurial cells varied from 20 to 60% between neoplasms. Thus, it is necessary to examine multiple tumor sections to accurately detect the claudin-1 expression.

In conclusion, despite the rarity of reported cases in veterinary medicine, perineurioma should be considered as a potential differential diagnosis for masses associated with peripheral nerves. In cases of suspected canine intraneural perineuriomas, confirmation of the diagnosis can be achieved by identifying characteristic histologic pseudo-onion bulb structures and observing positive immunolabeling for laminin and claudin-1 in perineurial cells. The peculiar histomorphology of perineuriomas generally distinguishes them from other nerve sheath tumors, such as schwannomas. While both perineuriomas and schwannomas exhibit laminin expression, the application of claudin-1 and Sox-10 may aid additional specificity in identifying cell origin.

## Data availability statement

The original contributions presented in the study are included in the article/Supplementary material, further inquiries can be directed to the corresponding author.

## Ethics statement

Ethical approval was not required for the studies involving animals in accordance with the local legislation and institutional requirements because the dog was submitted for standard necropsy to the Department of Pathobiology and is not subject to animal ethic guideline. Written informed consent was obtained from the owners for the participation of their animals in this study. Written informed consent was obtained from the participant/patient(s) for the publication of this case report.

## Author contributions

J-HY drafted the manuscript. BS, TJ, AY, and MH participated in the clinical management. BS, TJ, and AY performed neurologic examinations. MH interpreted the findings of diagnostic imaging and provided imaging figures. J-HY and MS performed postmortem examinations (gross and histopathology) and provided gross and histopathologic figures. AM, LS, and AH provided unpublished cases. BS, TJ, AY, MH, AM, LS, AH, and MS participated in the revision of the manuscript. All authors contributed to the article and approved the submitted version.
