# Supplementary material for: Case report: Intraneural perineurioma in dogs: a case series and brief literature review
Source: Front Vet Sci. 2024 Jan 11;10:1233230. doi: 10.3389/fvets.2023.1233230 (PMC10808598; doi:10.3389/fvets.2023.1233230)
Supplement: Supplementary file 1 [file Table_1.docx]

| Vimentin | Laminin | Claudin-1 | S100 | GFAP | Sox-10 | Periaxin | Collagen IV | GLUT-1 | Neurofila-ment | EMA | Reference |
| --- | --- | --- | --- | --- | --- | --- | --- | --- | --- | --- | --- |
| + | + | N/A | - | N/A | N/A | N/A | - | N/A | N/A | N/A | Higgins et al., 2006 |
| + | N/A | N/A | - | N/A | N/A | N/A | N/A | N/A | N/A | - | Martins et al., 2010 |
| N/A | + | + | - | N/A | N/A | N/A | N/A | - | - | N/A | Cornelis et al., 2012 |
| N/A | + | N/A | + | + | Inconclusive | + | N/A | N/A | N/A | N/A | Sisó et al. 2023^1^ |
| N/A | + | N/A | + | + | + | + | N/A | N/A | N/A | N/A | Sisó et al. 2023^2^ |
| N/A | + | N/A | N/A | N/A | + | + | N/A | N/A | N/A | N/A | Sisó et al. 2023^3^ |
| + | + | + | - | - | - | - | N/A | N/A | N/A | N/A | Cornell University, 2007 |
| + | + | + | + | - | - | - | N/A | N/A | N/A | N/A | Texas A&M University, 2019 |
| + | + | + | - | - | - | - | N/A | N/A | N/A | N/A | Auburn University, 2019 |

**Supplement Table 1. Various antigens expressed by neoplastic perineurial cells in canine perineuriomas.**

**EMA: Epithelial membrane antigen; GFAP: Glial fibrillary acidic protein; N/A: Not available, + Expressed, - Not expressed.**

**^1, 2, 3^ denotes the three cases used in the case-series (14).**

| **Primary antibody** | **Host** | **Source** | **Catalog** | **Dilution (primary antibody)** | **Antigen retrieval** | **Detection** |
| --- | --- | --- | --- | --- | --- | --- |
| Vimentin | Mouse | Biocare | PM 048 AA | Ready to use | DIVA | DAB |
| S100 | Rabbit | DAKO | Z0311 | 1: 1000 | DIVA | DAB |
| GFAP | Rabbit | Biocare | PP040AA | Ready to use | DIVA | DAB |
| Periaxin | Rabbit | Sigma-Aldrich | HPA001868 | 1:1000 | Bond Epitope Retrieval Solution | DAB |
| Sox-10 | Rabbit | Abcam | ab18086 | 1:500 | Heat-induced epitope retrieval | DAB |
| NF200 | Mouse | Novocastra (Leica) | NCL-NF200 | 1:50 | Citrate | Vector NovaRED |
| Laminin | Rabbit | Biogenex | PU078-UP | 1:50 | Protease | Vector NovaRED |

**Supplement Table 2. Details of various markers used for immunohistochemical profiling of canine intraneural perineuriomas (case 1-3).**

**DAB= 3,3-diaminobenzidine; GFAP: Glial fibrillary acidic protein**

Immunohistochemistry staining was performed on all formalin-fixed paraffin-embedded tissues from canine perineuriomas (cases 1-3). The tissues, sectioned at 5 µm, underwent deparaffinization, antigen retrieval pretreatment, and blocking of endogenous peroxidase activity. Primary antibodies, tailored to specific immunomarkers (vimentin, S100, GFAP, Periaxin, Sox-10, NF200, laminin, and claudin-1), were appropriately diluted and applied to the slides. Following incubation with secondary antibodies and 3,3-diaminobenzidine (DAB) chromogen, the slides were counterstained with hematoxylin, dehydrated, cleared, and mounted.
